# Prescription rate and treatment patterns for allergic rhinitis from 2010 to 2018 in South Korea: a retrospective study

**DOI:** 10.1186/s12948-021-00158-5

**Published:** 2021-10-11

**Authors:** Jaemin Son, Eun-San Kim, Hee-seung Choi, In-Hyuk Ha, Donghyo Lee, Yoon Jae Lee

**Affiliations:** 1 Jaseng Hospital of Korean Medicine, 536 Gangnam-daero, Gangnam-gu, Seoul, 06110 Republic of Korea; 2 Jaseng Spine and Joint Research Institute, Jaseng Medical Foundation, 3F, 538 Gangnam- daero, Gangnam-gu, Seoul, 06110 Republic of Korea; 3 Department of Medical Science of Meridian, College of Korean Medicine, Graduate School, Kyung Hee University, 02453 Seoul, Republic of Korea; 4 Department of Ophthalmology, Otolaryngology, and Dermatology, College of Korean Medicine, Woo-Suk University, 55338 Jeonju, Republic of Korea

**Keywords:** Allergic rhinitis, Medication, Prescription pattern, Prescription trend

## Abstract

**Background:**

There has been little investigation on how guidelines for allergic rhinitis (AR) treatment are applied in current clinical practice. We aimed to analyze prescription trends and patterns for AR treatment according to patient characteristics over a 9-year period in Korea.

**Methods:**

We used cross-sectional data from the Korean Health Insurance Review & Assessment Service National Patient Sample from 2010 to 2018. We analyzed 1,719,194 patients with AR as the principal diagnosis. Prescription rates of antihistamines, steroids, and other drugs; combination prescriptions; and first-choice prescriptions were analyzed.

**Results:**

The prescription rate of first-generation antihistamines decreased over the years (2010: 29.13; 2018: 23.41). By contrast, the prescription rate of systemic steroids (2010: 23.60; 2018: 28.70), nasal steroids (2010: 9.70; 2018: 14.67), and leukotriene receptor antagonists (LTRAs) (2010: 11.13; 2018: 26.56) increased. The prescription rate of steroids was lower in patients aged 0–5 years and ≥ 65 years than in other age groups and that of LTRAs was the highest in patients aged 0–5 years. The rate of combination prescribing antihistamines and nasal steroids increased (2010: 7.99; 2018: 12.09). The rate of first-choice prescriptions with antihistamines and nasal steroids also increased (2010: 4.72; 2018: 7.24).

**Conclusions:**

The results confirmed a decrease in antihistamine prescriptions, especially with first-generation, and an increase in steroid and LTRA prescriptions in patients with AR in Korea. Regarding prescription patterns, steroids were increasingly prescribed in combination with antihistamines. However, the trend was opposite in the 0–5 years and ≥ 65 years groups.

**Supplementary Information:**

The online version contains supplementary material available at 10.1186/s12948-021-00158-5.

## Background

Allergic rhinitis (AR) is a common disease worldwide [1]. In particular, pediatric AR has a high incidence rate, with a global prevalence of approximately 14% at the ages of 13 and 14 years [2]. In 2018, 16.7 % of adults in South Korea were diagnosed with AR [3]. The disease reduces workers’ productivity [4] and negatively affects the quality of sleep [5] and quality of life [6]. It can also cause complications such as sinusitis or otitis media [7].

Allergic Rhinitis and its Impact on Asthma (ARIA) guidelines recommend second-generation antihistamines and nasal steroids as medications for AR. Leukotriene receptor antagonists (LTRAs) are also recommended [8]. AR-related clinical guidelines developed in South Korea recommend the use of second-generation antihistamines, LTRAs, and nasal steroids [9]. However, studies have mainly focused on the treatment efficacy through randomized controlled trials, and few studies have assessed how different medications are used in clinical practice [10–13]. Ghouri N, et al. [10] studied prescription of antihistamines and drugs used in nasal allergy from 2001 to 2005 in the United Kingdom (UK). However, guidelines have since changed [8] and the types of medications have varied. Subsequently, a systematic survey of physicians was conducted in Italy [12] and the UK [11]. There were differences in prescription patterns depending on the region, indicating that research on prescription patterns in Korea is also necessary. Although a survey on prescription patterns was conducted with physicians in Korea [13], the samples used in that study were limited in terms of representing the entire Korean population due to the nature of the study design. In addition, it is difficult to analyze long-term trends from previous studies due to the nature of cross-sectional studies, and it was difficult to identify prescription patterns according to patient characteristics. Therefore, this study was aimed at analyzing medication prescription trends for AR and prescription patterns based on patient characteristics using data from a nationwide insurance claims database over a span of 9 years.

## Methods

### Data

Data from the Korean Health Insurance Review & Assessment Service National Patient Sample (HIRA-NPS) from 2010 to 2018 were used in this study. HIRA-NPS consists of cross-sectional data constructed using stratified sampling of 3% of all patients who used medical institutional services each year according to sex and age group (5-year interval). Data on approximately 1.45 million patients are extracted every year, and the data can be accessed through the de-identification of personally identifiable information. HIRA-NPS is a single national health insurance database that represents the South Korean population, and the data therein have high validity in terms of representativeness [14]. The database includes demographic characteristics such as patient sex and age, diagnosis code, medical procedures provided to patients by medical institutions, and details of medication and associated costs.

### Population and characteristics

The definition of AR was set according to the Korean Standard Classification of Diseases, 7th Revision. Therefore, AR patients were defined as patients for whom AR was recorded as the principal diagnosis (codes: J30, J301, J302, J303, and J304) at least once per year. The patient characteristics extracted from the data included sex, age, and comorbidity. In terms of age, patients were classified into six age groups: 0–5 years, 6–12 years, 13–19 years, 20–39 years, 40–64 years, and ≥ 65 years. To determine the comorbidity status of other allergic diseases, if the disease was recorded as a principal or secondary diagnosis at least once in a given year, the patient was considered to have the disease. Types of comorbidities included allergic asthma (code J450) and allergic dermatitis (codes L20, L208, L2080, L2088, and L209).

### Measures

For the analysis of the type of medication prescribed, the major drugs for AR treatment were selected from literature, like the ARIA guidelines [8, 15–17]. Accordingly, antihistamines, steroids, LTRAs, systemic decongestants, and cough and cold preparations were included in the analysis. Antihistamines were divided into first- and second-generations with total prescriptions; steroids were also divided into total, nasal, and systemic steroids. Medication types were defined according to the Anatomical Therapeutic Chemical Classification System (see Additional file 1: Table S1).

Next, 1-year episodes in patients with AR were analyzed to examine patients’ prescription patterns. First, prescription combination patterns were analyzed. Cases were classified as prescription of first-generation antihistamines only, second-generation antihistamines only, antihistamines and nasal steroids, or nasal steroids only. Furthermore, to examine the first-choice prescription for AR patients, we investigated patient prescriptions for the initial episodes. Cases were classified as prescription of antihistamines only, nasal steroids only, or antihistamines and nasal steroids. The prescription of LTRAs, decongestants, and cough and cold preparations was not considered in the first-choice prescription analysis.

### Analysis

Basic patient characteristics included the number of patients (*n*) and percentages, and a chi-square test was performed to analyze differences in characteristics by year. Annual prescription rates were investigated by medication type. Furthermore, the prescription rate was presented as per 100 patients with AR and was sex- and age-standardized according to the population composition in 2010. To determine the trend in prescription rate for each year, the p trend was analyzed by linear regression analysis, considering the year as a continuous variable. Statistical significance was set at *P*-value ≤ 0.05.

A subgroup analysis according to patient characteristics was performed with age (0–5, 6–12, 13–19, 20–39, 40–64, and ≥ 65 years) and the comorbidity status of allergic asthma and allergic dermatitis. The age groups of 0–5 years and ≥ 65 years had the most pronounced differences in pattern from the total population. The results for other subgroups are presented in Additional file 1: Table S2a–h.

## Results

From 2010 to 2018, a total of 1,719,194 AR patients were included in the study, of which 794,726 (46.22%) were male and 924,468 (53.77%) were female patients. The basic characteristics of patients for each year are shown in Additional file 1: Table S3. The number of AR patients by year showed a year-on-year increase (*n*, 2010: 167,524; 2018: 213,420). The proportion of patients aged 0–5 years increased (percentage, 2010: 14.0%; 2018: 17.5%, *P* < 0.001), while that of patients aged 13–19 years decreased (percentage, 2010: 10.5%; 2018: 8.1%, *P* < 0.001). Furthermore, the proportion of patients with allergic asthma and allergic dermatitis increased in general (percentage of allergic asthma patients, 2010: 8.1%; 2018: 10.3%, *P* < 0.001; percentage of allergic dermatitis patients, 2010: 9.3%; 2018: 10.4%, *P* < 0.001).

**Fig. 1:**
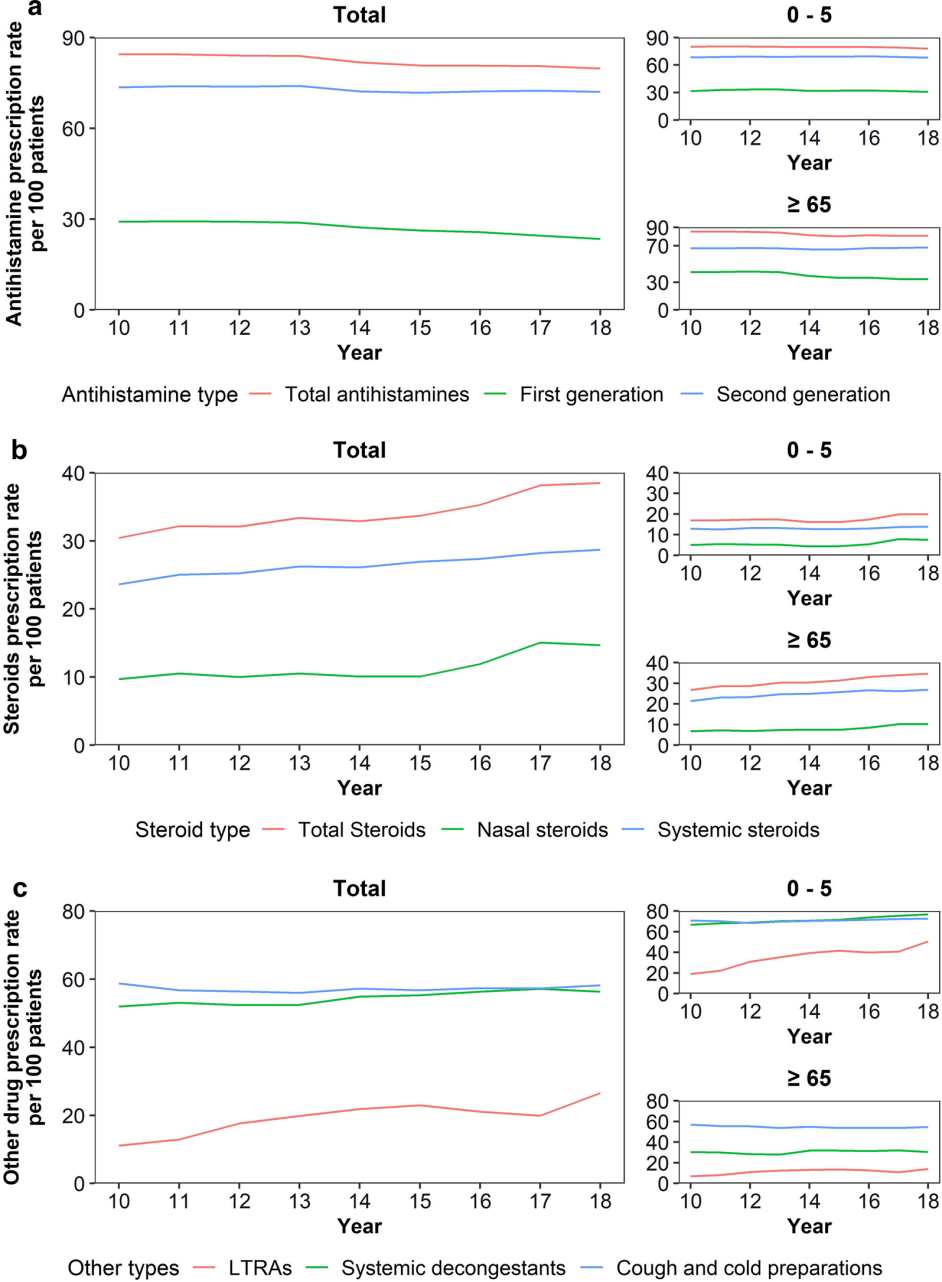
Rate of prescription of antihistamines, steroids, and other drugs by year. Prescription rates per 100 patients are presented by year. **a** Antihistamines; **b** Steroids; **c** Other drugs (LTRAs, other nasal preparations, and cough and cold preparations). Rates were sex- and age-standardized according to the population composition of 2010. LTRAs: leukotriene receptor antagonists

The prescription rate was generally higher for second-generation antihistamines than for first-generation antihistamines (First-generation antihistamine prescription rate, 2010: 29.13; 2018: 23.41, *P* < 0.001; Second-generation antihistamine prescription rate, 2010: 73.56; 2018: 72.02, *P* = 0.010; Table 1). Moreover, the prescription rate for first-generation antihistamines showed a more steeply decreasing trend (prescription rate, 2010: 29.13; 2018: 23.41, *P* < 0.001) than first-generation antihistamines. In particular, the prescription rate for first-generation antihistamines in the age group ≥ 65 years markedly decreased (prescription rate, 2010: 41.15; 2018: 33.39, *P* < 0.001; Fig. 1a).

**Table 1:** Rates of prescription and pattern by year

Year	2010	2011	2012	2013	2014	2015	2016	2017	2018	Beta	*P*-value
Antihistamines											
Total antihistamines	84.47	84.44	84.05	83.87	81.81	80.77	80.70	80.53	79.75	-0.67	< 0.001
First generation	29.13	29.25	29.12	28.78	27.27	26.25	25.70	24.56	23.41	-0.77	< 0.001
Second generation	73.56	73.89	73.78	73.97	72.20	71.77	72.20	72.46	72.02	-0.26	0.010
Steroids											
Total steroids	30.43	32.17	32.09	33.38	32.90	33.69	35.28	38.17	38.50	0.95	< 0.001
Nasal steroids	9.70	10.51	10.01	10.54	10.09	10.07	11.90	15.08	14.67	0.61	0.008
Systemic steroids	23.60	25.04	25.25	26.24	26.12	26.94	27.36	28.21	28.70	0.58	< 0.001
Other drugs											
Leukotriene antagonists	11.13	12.88	17.58	19.86	21.85	22.98	21.08	19.91	26.56	1.55	0.002
Systemic decongestants	51.97	53.05	52.37	52.46	54.91	55.32	56.36	57.13	56.34	0.68	< 0.001
Cough and cold preparations	58.79	56.71	56.43	55.99	57.23	56.77	57.36	57.31	58.17	0.03	0.791
Prescription combination pattern											
First-generation antihistamines only	10.50	10.08	9.83	9.43	9.19	8.60	8.12	7.59	7.28	-0.41	< 0.001
Second-generation antihistamines only	49.46	48.96	49.04	48.86	48.53	48.52	47.71	46.57	47.14	-0.32	< 0.001
Antihistamines and nasal steroids	7.99	8.74	8.28	8.72	8.32	8.28	9.83	12.50	12.09	0.51	0.009
Nasal steroids only	1.71	1.77	1.73	1.82	1.77	1.79	2.06	2.58	2.58	0.11	0.004
First-choice prescription											
Antihistamines only	76.33	75.76	75.27	74.68	72.35	71.1	70.06	68.04	67.27	-1.22	< 0.001
Nasal steroids only	1.94	1.99	1.93	2.06	1.95	1.96	2.21	2.92	2.90	0.12	0.011
Antihistamines and nasal steroids	4.72	5.15	4.88	5.22	4.97	4.94	5.69	7.42	7.24	0.30	0.008

Prescription and pattern rates per 100 patients are presented by year. Rates were sex- and age-standardized according to the population composition of 2010

The prescription rate was generally higher for systemic steroids than for nasal steroids and showed an increasing trend (Nasal steroids prescription rate, 2010: 9.70; 2018: 14.67, *P* = 0.008; Systemic steroids prescription rate, 2010: 23.60; 2018: 28.70, *P* < 0.001). In particular, the prescription rate for nasal steroids has increased significantly since 2015 (prescription rate, 2015: 10.07; 2018: 14.67). The prescription rate for total steroids was the lowest in patients aged 0–5 and ≥ 65 years; however, the rate increased over time for patients aged ≥ 65 years (0–5-year prescription rate, 2010: 16.87; 2018: 19.88; *P* = 0.072; ≥65 years prescription rate, 2010: 26.76; 2018: 34.64; *P* < 0.001) (Fig. 1b). The steroid prescription rate in asthma patients was higher than that in atopic dermatitis patients, and the prescription rate increased in both groups (prescription rate for asthma, 2010: 30.87; 2018: 37.30; *P* = 0.001; prescription rate for atopic dermatitis, 2010: 26.70; 2018: 32.96; *P* = 0.002) (see Additional file 1: Table S2g–h).

The prescription rate of LTRAs has increased, with the largest rate of increase in 2018 (prescription rate, 2010: 11.13; 2018: 26.56; *P* < 0.001). The prescription rate for systemic decongestants increased (prescription rate, 2010: 51.97; 2018: 56.34; *P* < 0.001), while that for cough and cold preparations did not show a significant change (prescription rate, 2010: 58.79; 2018: 58.17; *P* = 0.791). The prescription rate of LTRAs, systemic decongestants, and cough and cold preparations were the highest in patients aged 0–5 years and showed a decreasing trend with age. Furthermore, the prescription rate was higher for cough and cold preparations than for systemic decongestants in patients aged ≥ 65 years. The increase in the prescription rate of LTRAs was the highest in the age group 0–5 years (prescription rate, 2010: 19.05; 2018: 50.48; *P* < 0.001) (Fig. 1c). Also, the largest difference in prescription rate between allergic asthma and atopic dermatitis patients and total population was LTRAs (prescription rate in allergic asthma patient, 2010: 16.69; 2018: 41.34; P < 0.001; prescription rate in atopic dermatitis patient, 2010: 26.25; 2018: 45.91; P < 0.001) (see Additional file 1: Table S2g–h).

**Fig. 2:**
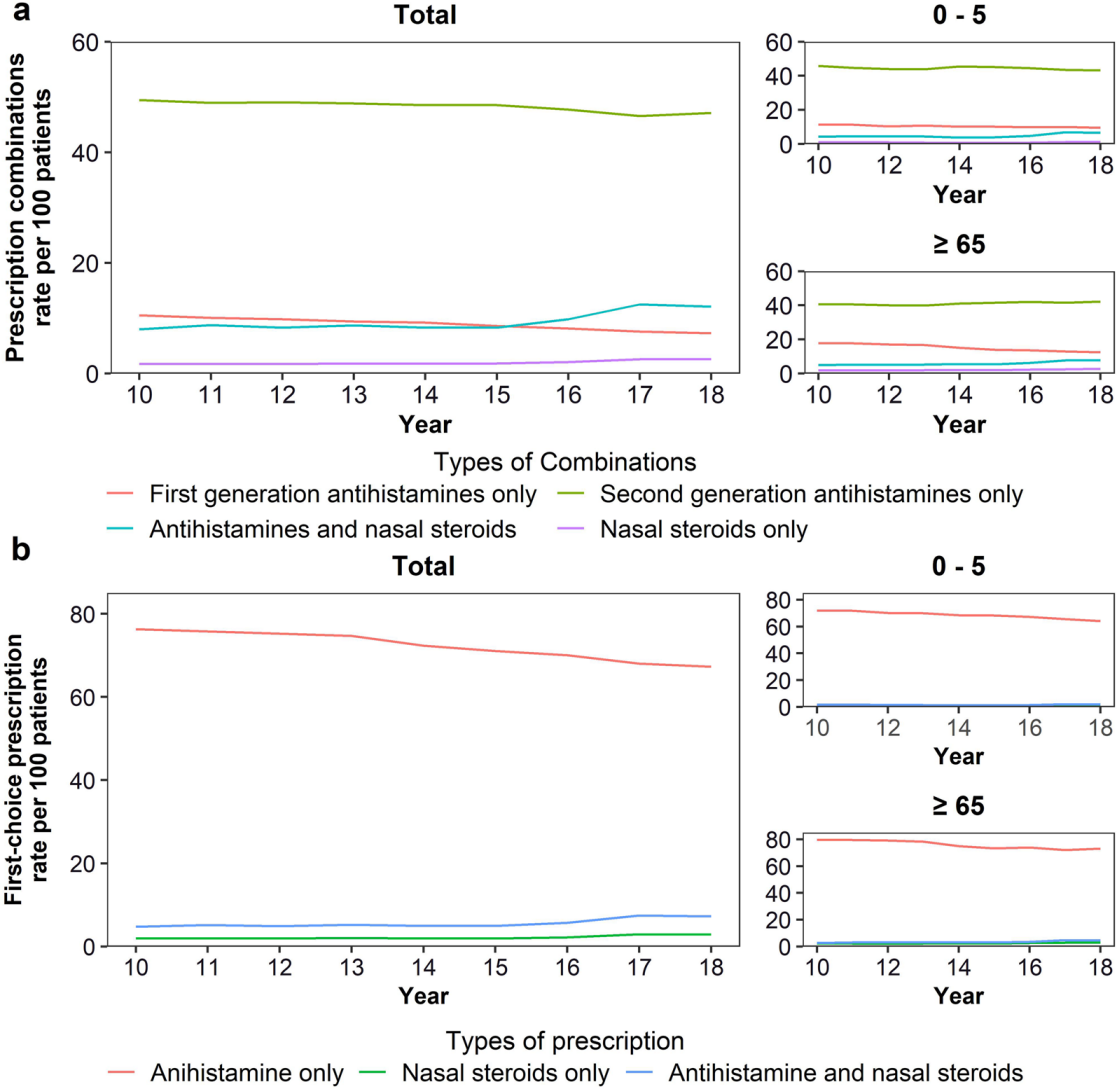
Rates of prescription pattern by year. The prescription rate per 100 patients is presented by year. **a** Combination patterns; **b** First-choice prescription. An episode refers to all medical records of the patient receiving allergic rhinitis treatment for 1 year. Rates were sex- and age-standardized according to the population composition of 2010

The prescription rate of “second-generation antihistamines only” was the highest overall; however, it showed a decreasing trend (prescription rate, 2010: 49.46; 2018: 47.14, *P* < 0.001). By contrast, the prescription rate of “antihistamines and nasal steroids” (prescription rate, 2010: 7.99; 2018: 12.09, *P* = 0.009) and “nasal steroids only” showed an increasing trend (prescription rate, 2010: 1.71; 2018: 2.58, *P* = 0.004). The trend was generally consistent in all age groups. Nevertheless, the prescription rate of “antihistamines and nasal steroids” and “nasal steroids only” was lower in the age groups 0–5 years and ≥ 65 years than in the other groups (Fig. 2a).

“Antihistamines only” was the most often used first-choice prescription category; however, the corresponding prescription rate decreased over time (prescription rate, 2010: 76.33; 2018: 67.27, *P* < 0.001). By contrast, the prescription rate of combination prescription of antihistamines and nasal steroids (prescription rate, 2010: 4.72; 2018: 7.24, *P* = 0.008) and “nasal steroids only” (prescription rate, 2010: 1.94; 2018: 2.90, *P* = 0.011) increased. The trend was consistent in most age groups; nevertheless, the prescription rate of combination prescription of antihistamines and nasal steroids (prescription rate, 2010: 1.54; 2018: 1.90, *P* = 0.548) and “nasal steroids only” (prescription rate, 2010: 0.98; 2018: 1.22, *P* = 0.728) was the lowest in patients aged 0–5 years, and there was no significant increase in these rates (Fig. 2b).

## Discussion

In this study, the trends and patterns of medication prescriptions for patients with AR were analyzed for the period from 2010 to 2018. The results confirmed that the prescription of antihistamines, particularly first-generation antihistamines, decreased while the prescription of steroids and LTRAs increased. The prescriptions of “antihistamines only” decreased, whereas the prescription rate of “antihistamine and nasal steroids” and “nasal steroids only” increased. However, the trends differed depending on patient characteristics. In particular, the increase in the rate of steroid prescription was relatively low in patients aged 0–5 years and ≥ 65 years. Furthermore, for these age groups, the rate of steroid prescription was also relatively low in combination prescriptions and first-choice prescriptions.

Some results of this study are consistent with a previously conducted survey in Korea. Antihistamines were the first-choice medication. Furthermore, pediatricians have reported lower prescription rates of combinations of antihistamines and nasal steroids and higher prescription rates of LTRAs than the prescription rates reported by physicians of other specialties [13]. This trend was also confirmed in a subgroup analysis of our study. However, the previous study showed that the combination of nasal steroids and antihistamines was the most prescribed. By contrast, in the present study, the prescription rate of second-generation antihistamines alone was the highest, although it showed a decreasing trend. This variance may be attributed to difference in study subjects. In the present study, medical records of all relevant patients were analyzed, and thus, patients with mild AR were also included in the analysis. By contrast, as the previous study performed a survey that relied upon the physician’s memory, only prescription patterns for patients with frequent visits may have been reported.

In particular, a remarkable decrease was noted in the prescription rate of first-generation antihistamines, which could be attributed to the superiority of second-generation antihistamines. The latter have a faster and longer-lasting effect than the first-generation antihistamines [18], and are safer with only a slight sedative effect [19]. Furthermore, second-generation antihistamines have been reported to be safer than first-generation antihistamines even when used in combination with other drugs [20]. Also, ARIA and standard guidelines of the Korean Academy of Asthma, Allergy, and Clinical Immunology recommend the use of second-generation antihistamines [8, 9].

The use of nasal steroids has been increasing. Moreover, the combination of nasal steroids and antihistamines has shown the largest rate of prescription increase. ARIA and the standard guidelines of the Korean Academy of Asthma, Allergy, and Clinical Immunology state that nasal steroids are more effective than antihistamines in relieving nasal blockage [8, 9]. These results, and changes in the guidelines, are thought to have had some effect on the prescription patterns. However, compared with the extensive use of nasal steroids in other countries [11, 12], the use of nasal steroids in Korea has remained at around 10–20 %.

Rather, it should be noted that the use of systemic steroids was the highest among steroids in Korea, and the use of systemic steroids has shown an increasing trend. Prescription rates for systemic steroids were also the highest for the age groups 0–5 years and ≥ 65 years. Systemic steroids are only recommended for patients with very severe and therapy-resistant symptoms due to concerns of adverse events [21]. This pattern was not identified in other countries [11, 12], and was not reported in the previous survey in Korea [13]. Further studies are required to investigate the characteristics and prognosis of patients prescribed these drugs.

We observed increased prescription of LTRAs. These drugs alleviate nasal and ocular symptoms [22] and are more effective in combination therapy with antihistamines and steroids [23–25]. In particular, the safety of LTRAs demonstrated in children [26, 27] may have resulted in the increased prescription of LTRAs in patients aged 0–5 years. Based on these results, the reimbursement criteria for LTRAs were expanded in Korea at the end of 2017. Before the amendment, reimbursement was only possible when there was no improvement with antihistamine treatment, but now LTRAs are reimbursed even when prescribed as a first-line treatment [28]. The increase in LTRAs prescriptions in 2018 is thought to have reflected these changes.

The prescription rates for allergic asthma and atopic dermatitis patients were similar to the total allergic rhinitis patients, except that the prescription rate of LTRAs was higher for allergic asthma and atopic dermatitis patients. LTRAs are effective for controlling asthma symptoms [29]. Therefore, physicians might have prescribed the LTRAs for allergic rhinitis patients with asthma symptoms. Although the evidence of using LTRAs for atopic dermatitis is limited [30], the high prescription rates might be due to the younger age of atopic dermatitis patients (data not presented). The prescription rates of other medications should be interpreted with caution. Depending on patient’s severity, other medications, especially steroids, might have been prescribed with allergic asthma or atopic dermatitis as primary diagnosis, not allergic rhinitis. If the patient had been prescribed steroids with allergic asthma or atopic dermatitis as primary diagnosis closely before the patient visited for allergic rhinitis, the physicians might have not prescribed the steroids. Considering this, the actual prescription rates of steroids might be higher than observed.

Our study has limitations for generalization [31]. First, this study analyzed insurance-covered medications prescribed in clinical practice, therefore non-covered medications were not included in the analysis. In particular, xylometazoline hydrochloride, the topical decongestant with the highest sales, could not be analyzed in this study because it is a non-reimbursable medicine. Accordingly, for decongestants, only systemic decongestants were investigated, and in this regard, generalizability is a drawback of this study. Also, we did not investigate antihistamines and topical decongestants sold as OTC preparations. This does not serve as a bias because this study aimed to investigate only the prescriptions of the clinicians. However, as OTC preparations are thought to have affected the prescription trend, the exact description of the factors related to the prescription trend remains a limitation. Further, allergen immunotherapy is a treatment emphasized in the guidelines and needs to be analyzed. However, since it is not covered by the national health insurance of South Korea, it could not be included in this study. In addition, there was a bias in the episode analysis of patients because yearly cross-sectional data were used. In particular, when analyzing first-choice prescriptions, if the patient’s prescription continued from the previous year, the prescription information of the previous year was not considered.

Nevertheless, to the best of our knowledge, the present study is significant because it is the first most extensive analysis on the long-term trends of medication prescriptions to treat AR. Furthermore, actual clinical decisions were analyzed from various perspectives with an analysis of a range of medication prescriptions and patterns. In particular, nationwide data representing the South Korean population were used, and all citizens and medical institutions are members of the Korean health insurance system as a regulatory requirement. Thus, the data were highly representative, and there were few limitations pertaining to the generalization of the study findings. Since there have been few analyses of AR prescription trends, the results of this study can be used to develop a clinical guideline in the future and can help confirm whether the guideline developed is clinically applicable.

## Conclusions

This study revealed a decrease in the prescriptions of antihistamines, especially first-generation antihistamines, and an increase in the prescriptions of steroids and LTRAs for patients with AR in South Korea. Furthermore, the rate of prescription of combinations of antihistamines and steroids increased. However, this trend differed depending on patient characteristics, and the rate of increase in steroid prescriptions was relatively lower in patients aged 0–5 years and ≥ 65 years. It is expected that these results can serve as basic research data for clinicians and policymakers in the development and application of relevant guidelines.

## Supplementary Material

**Additional file 1.** Supplemental materials.

## Data Availability

The Health Insurance Review & Assessment Service National Patient Sample (HIRA-NPS) is provided by the Health Insurance Service & Assessment Service in Korea. To protect privacy, access to the data is available only for certified researchers in South Korea.

## Abbreviations

AR: Allergic rhinitis
LTRAs: Leukotriene receptor antagonists
ARIA: Allergic Rhinitis and its Impact on Asthma
HIRA-NPS: Health Insurance Review & Assessment Service National Patient Sample
