# Supplementary material for: Prescription rate and treatment patterns for allergic rhinitis from 2010 to 2018 in South Korea: a retrospective study
Source: Clin Mol Allergy. 2021 Oct 11;19:20. doi: 10.1186/s12948-021-00158-5 (PMC8504056; doi:10.1186/s12948-021-00158-5)
Supplement: Supplementary file 1 — Additional file 1. Supplemental materials. [file 12948_2021_158_MOESM1_ESM.docx]

**SUPPLEMENTAL MATERIAL**

Supplemental Table 1. Types of medication according to the Anatomical Therapeutic Chemical Classification System

| **Type** | **Sub-type** | **ATC code** | |
| --- | --- | --- | --- |
| **Antihistamine** | First generation | N05BB01 | Hydroxyzine |
|  |  | R06AA02 | Dimenhydrate |
|  |  | R06AA04 | Clemastine |
|  |  | R06AA07 | Diphenylpyraline |
|  |  | R06AB04 | Chlorphenamine |
|  |  | R06AE06 | Oxatomide |
|  | Second generation | R06AD07 | Mequitazine |
|  |  | R06AE07 | Cetirizine |
|  |  | R06AE09 | Levocetirizine |
|  |  | R06AX13 | Loratadine |
|  |  | R06AX17 | Ketotifen |
|  |  | R06AX19 | Azelastine |
|  |  | R06AX22 | Ebastine |
|  |  | R06AX24 | Epinastine |
|  |  | R06AX25 | Mizolastine |
|  |  | R06AX26 | Fexofenadine |
|  |  | R06AX27 | Desloratadine |
|  |  | R06AX28 | Rupatadine |
|  |  | R06AX* | Bepotastine |
|  |  | R06AX* | Olopatadine |
| **Steroid** | Nasal steroids | R01AD | Corticosteroids |
|  |  | R01AD01 | Beclometasone |
|  |  | R01AD03 | Dexamethasone |
|  |  | R01AD05 | Budesonide |
|  |  | R01AD08 | Fluticasone |
|  |  | R01AD09 | Mometasone |
|  |  | R01AD11 | Triamcinolone |
|  |  | R01AD12 | Fluticasone furoate |
|  |  | R01AD58 | Fluticasone, combinations |
|  | Systemic steroids | H02AB01 | Betamethasone |
|  |  | H02AB02 | Dexamethasone |
|  |  | H02AB04 | Methylprednisolone |
|  |  | H02AB06 | Prednisolone |
|  |  | H02AB08 | Triamcinolone |
|  |  | H02AB09 | Hydrocortisone |
|  |  | H02AB13 | Deflazacort |
| **Leukotriene receptor antagonist** | | R03DC01 | Zafirlukast |
|  |  | R03DC02 | Pranlukast |
|  |  | R03DC03 | Montelukast |
|  |  | R03DC53 | Montelukast, combinations |
| **Other nasal preparations** | | R01B | Nasal decongestants for systemic use |
| **Cough and cold preparations** | | R05 | Cough and cold preparations |
| * Drugs not assigned ATC code were analyzed using the name of the drug. | | | |

Supplemental Table 2a. Rates of prescription and patterns by year for age 0–5 years

| **Year** | **2010** | **2011** | **2012** | **2013** | **2014** | **2015** | **2016** | **2017** | **2018** | **Beta** | ***P*-value** |
| --- | --- | --- | --- | --- | --- | --- | --- | --- | --- | --- | --- |
| **Age 0-5 years** | | | | | | | | | | | |
| **Antihistamines** | | | | | | | | | | | |
| Total antihistamines | 79.92 | 80.48 | 80.07 | 79.89 | 79.53 | 79.48 | 79.54 | 79.21 | 77.92 | -0.22 | 0.005 |
| First generation | 31.45 | 32.81 | 33.33 | 33.42 | 31.84 | 31.99 | 32.18 | 31.39 | 30.75 | -0.18 | 0.132 |
| Second generation | 68.36 | 68.9 | 69.41 | 68.85 | 69.24 | 69.18 | 69.52 | 68.88 | 68.05 | -0.01 | 0.856 |
| **Steroids** | | | | | | | | | | | |
| Total steroids | 16.87 | 17.04 | 17.36 | 17.32 | 16.15 | 16.09 | 17.32 | 19.84 | 19.88 | 0.32 | 0.072 |
| Nasal steroids | 4.98 | 5.52 | 5.2 | 5.12 | 4.36 | 4.41 | 5.39 | 7.88 | 7.58 | 0.29 | 0.077 |
| Systemic steroids | 12.92 | 12.61 | 13.25 | 13.19 | 12.77 | 12.7 | 13.03 | 13.72 | 13.92 | 0.11 | 0.06 |
| **Other drugs** | | | | | | | | | | | |
| Leukotriene antagonists | 19.05 | 22.18 | 30.9 | 35.32 | 39.36 | 41.73 | 39.78 | 40.85 | 50.48 | 3.43 | < 0.001 |
| Systemic decongestants | 66.85 | 68.14 | 68.83 | 70.18 | 70.73 | 71.67 | 73.81 | 75.53 | 76.75 | 1.22 | < 0.001 |
| Cough and cold preparations | 70.94 | 70.23 | 68.3 | 69.77 | 70.54 | 71.01 | 71.7 | 72.34 | 72.82 | 0.36 | 0.026 |
| **Prescription combination pattern** | | | | | | | | | | | |
| First-generation antihistamines only | 11.28 | 11.26 | 10.37 | 10.78 | 10.06 | 10.09 | 9.78 | 9.97 | 9.53 | -0.21 | < 0.001 |
| Second-generation antihistamines only | 45.75 | 44.73 | 44.01 | 43.85 | 45.41 | 45.12 | 44.5 | 43.51 | 43.11 | -0.2 | 0.08 |
| Antihistamines and nasal steroids | 4.13 | 4.6 | 4.35 | 4.3 | 3.77 | 3.82 | 4.66 | 6.86 | 6.44 | 0.27 | 0.052 |
| Nasal steroids only | 0.85 | 0.93 | 0.85 | 0.83 | 0.59 | 0.6 | 0.74 | 1.03 | 1.14 | 0.02 | 0.525 |
| **First-choice prescription** | | | | | | | | | | | |
| Antihistamines only | 72.03 | 71.8 | 70.16 | 69.86 | 68.59 | 68.2 | 67.22 | 65.59 | 64.15 | -0.96 | < 0.001 |
| Nasal steroids only | 0.98 | 1.08 | 1 | 1.02 | 0.7 | 0.67 | 0.78 | 1.23 | 1.22 | 0.01 | 0.728 |
| Antihistamines and nasal steroids | 1.54 | 1.85 | 1.51 | 1.4 | 1.18 | 1.18 | 1.38 | 2.09 | 1.9 | 0.03 | 0.548 |
| Prescription and pattern rates per 100 patients are presented by year. Rates were sex- and age-standardized according to the population composition of 2010. | | | | | | | | | | | |

Supplemental Table 2b. Rates of prescription and patterns by year for age 6–12 years

| **Year** | **2010** | **2011** | **2012** | **2013** | **2014** | **2015** | **2016** | **2017** | **2018** | **Beta** | ***P*-value** |
| --- | --- | --- | --- | --- | --- | --- | --- | --- | --- | --- | --- |
| **Age 6-12 years** | | | | | | | | | | | |
| **Antihistamines** | | | | | | | | | | | |
| Total antihistamines | 85.85 | 85.47 | 84.66 | 84.63 | 83.89 | 83.6 | 84.18 | 84.27 | 83.41 | -0.26 | 0.003 |
| First generation | 23.71 | 23.31 | 23.66 | 23.1 | 22.96 | 22.81 | 22.63 | 21.34 | 21.38 | -0.29 | < 0.001 |
| Second generation | 77.71 | 78.23 | 77.51 | 77.9 | 77.02 | 77.05 | 77.81 | 78.2 | 77.36 | -0.03 | 0.645 |
| **Steroids** | | | | | | | | | | | |
| Total steroids | 27.19 | 28.81 | 28.64 | 28.64 | 27.38 | 27.68 | 29.72 | 33.77 | 33.15 | 0.66 | 0.019 |
| Nasal steroids | 12.54 | 13.76 | 13.62 | 13.46 | 12.53 | 12.23 | 14.97 | 19.45 | 18.68 | 0.72 | 0.024 |
| Systemic steroids | 17.56 | 18.52 | 18.52 | 18.42 | 17.89 | 18.52 | 18.43 | 19.31 | 19.17 | 0.15 | 0.025 |
| **Other drugs** | | | | | | | | | | | |
| Leukotriene antagonists | 15.2 | 18.14 | 24.99 | 28.53 | 33.04 | 34.95 | 32.73 | 31.76 | 43.09 | 2.91 | < 0.001 |
| Systemic decongestants | 61.19 | 61.79 | 61.47 | 61.33 | 62.22 | 63.11 | 65.08 | 65.79 | 65.37 | 0.63 | < 0.001 |
| Cough and cold preparations | 60.96 | 59.45 | 58.33 | 57.82 | 60.4 | 59.89 | 61.71 | 60.67 | 60.43 | 0.17 | 0.314 |
| **Prescription combination pattern** | | | | | | | | | | | |
| First-generation antihistamines only | 7.54 | 6.64 | 6.57 | 6.19 | 6.35 | 6.08 | 5.86 | 5.4 | 5.47 | -0.23 | < 0.001 |
| Second-generation antihistamines only | 53.85 | 53.2 | 52.3 | 52.88 | 53.02 | 52.8 | 51.63 | 49.88 | 49.65 | -0.47 | 0.002 |
| Antihistamines and nasal steroids | 11.14 | 12.27 | 11.96 | 11.85 | 10.97 | 10.68 | 13.32 | 17.33 | 16.61 | 0.64 | 0.028 |
| Nasal steroids only | 1.4 | 1.5 | 1.66 | 1.61 | 1.56 | 1.54 | 1.65 | 2.12 | 2.08 | 0.07 | 0.007 |
| **First-choice prescription** | | | | | | | | | | | |
| Antihistamines only | 75.85 | 74.73 | 74.25 | 73.84 | 73.26 | 73 | 72.14 | 70.04 | 69.16 | -0.76 | < 0.001 |
| Nasal steroids only | 1.75 | 1.81 | 1.9 | 2.06 | 1.7 | 1.7 | 1.8 | 2.57 | 2.63 | 0.09 | 0.054 |
| Antihistamines and nasal steroids | 5.95 | 6.71 | 6.18 | 6.18 | 5.78 | 5.48 | 6.5 | 9 | 8.37 | 0.27 | 0.072 |
| Prescription and pattern rates per 100 patients are presented by year. Rates were sex- and age-standardized according to the population composition of 2010. | | | | | | | | | | | |

Supplemental Table 2c. Rates of prescription and patterns by year for age 13-19 years

| **Year** | **2010** | **2011** | **2012** | **2013** | **2014** | **2015** | **2016** | **2017** | **2018** | **Beta** | ***P*-value** |
| --- | --- | --- | --- | --- | --- | --- | --- | --- | --- | --- | --- |
| **Age 13-19 years** | | | | | | | | | | | |
| **Antihistamines** | | | | | | | | | | | |
| Total antihistamines | 85.56 | 84.82 | 85.06 | 84.77 | 82.22 | 80.98 | 80.85 | 81.03 | 80.82 | -0.71 | < 0.001 |
| First generation | 21.06 | 21.22 | 20.32 | 20.82 | 19.75 | 19.12 | 17.94 | 17.29 | 16.39 | -0.62 | < 0.001 |
| Second generation | 78.32 | 77.56 | 78.08 | 77.99 | 75.47 | 74.73 | 75.18 | 75.88 | 75.72 | -0.41 | 0.011 |
| **Steroids** | | | | | | | | | | | |
| Total steroids | 31.61 | 33.54 | 33.79 | 34.58 | 34.36 | 35.09 | 36.98 | 39.73 | 39.63 | 0.96 | < 0.001 |
| Nasal steroids | 11.14 | 12.4 | 12.25 | 12.74 | 12.23 | 12.22 | 14.37 | 17.68 | 17.26 | 0.73 | 0.004 |
| Systemic steroids | 24 | 24.92 | 25.49 | 26.02 | 26.14 | 26.88 | 27.6 | 28.12 | 28.24 | 0.53 | < 0.001 |
| **Other drugs** | | | | | | | | | | | |
| Leukotriene antagonists | 10.16 | 10.92 | 15.17 | 16.99 | 19.03 | 20.24 | 17.4 | 16.31 | 23.14 | 1.26 | 0.005 |
| Systemic decongestants | 57.15 | 57.75 | 57.41 | 57.07 | 60.47 | 60.8 | 60.93 | 62.13 | 60.59 | 0.63 | 0.002 |
| Cough and cold preparations | 56.41 | 52.39 | 52.67 | 52.2 | 53.36 | 53.62 | 53.41 | 54.04 | 55.24 | 0.05 | 0.786 |
| **Prescription combination pattern** | | | | | | | | | | | |
| First-generation antihistamines only | 6.91 | 6.92 | 6.53 | 6.31 | 6.31 | 5.95 | 5.38 | 4.76 | 4.76 | -0.29 | < 0.001 |
| Second-generation antihistamines only | 56.76 | 55.43 | 56.6 | 55.51 | 54.29 | 53.72 | 53.04 | 51.72 | 52.66 | -0.61 | < 0.001 |
| Antihistamines and nasal steroids | 9.46 | 10.41 | 10.47 | 10.68 | 10.39 | 10.12 | 12.07 | 14.89 | 14.29 | 0.59 | 0.005 |
| Nasal steroids only | 1.67 | 1.99 | 1.78 | 2.07 | 1.84 | 2.09 | 2.3 | 2.79 | 2.97 | 0.14 | 0.002 |
| **First-choice prescription** | | | | | | | | | | | |
| Antihistamines only | 76.51 | 75.41 | 75.44 | 74.59 | 72.05 | 70.93 | 69.6 | 67.54 | 67.33 | -1.26 | < 0.001 |
| Nasal steroids only | 1.86 | 2.14 | 2.04 | 2.27 | 2.08 | 2.22 | 2.45 | 3.12 | 3.25 | 0.15 | 0.002 |
| Antihistamines and nasal steroids | 6.18 | 6.67 | 6.81 | 7.24 | 6.68 | 6.5 | 7.72 | 9.58 | 9.25 | 0.37 | 0.007 |
| Prescription and pattern rates per 100 patients are presented by year. Rates were sex- and age-standardized according to the population composition of 2010. | | | | | | | | | | | |

Supplemental Table 2d. Rates of prescription and patterns by year for age 20–39 years

| **Year** | **2010** | **2011** | **2012** | **2013** | **2014** | **2015** | **2016** | **2017** | **2018** | **Beta** | ***P*-value** |
| --- | --- | --- | --- | --- | --- | --- | --- | --- | --- | --- | --- |
| **Age 20-39 years** | | | | | | | | | | | |
| **Antihistamines** | | | | | | | | | | | |
| Total antihistamines | 84.74 | 84.47 | 84.2 | 83.97 | 81.42 | 79.58 | 79.18 | 78.88 | 77.78 | -0.98 | < 0.001 |
| First generation | 26.65 | 26.38 | 26.29 | 25.81 | 24.18 | 22.81 | 21.81 | 20.85 | 18.88 | -0.99 | < 0.001 |
| Second generation | 75.31 | 75.27 | 75.14 | 75.07 | 72.92 | 71.75 | 71.97 | 72.12 | 71.5 | -0.57 | < 0.001 |
| **Steroids** | | | | | | | | | | | |
| Total steroids | 35.36 | 37.29 | 37.33 | 39.46 | 39.04 | 39.98 | 41.51 | 45.26 | 45.97 | 1.25 | < 0.001 |
| Nasal steroids | 10.78 | 11.38 | 10.32 | 11.59 | 11.25 | 11.15 | 13.25 | 16.58 | 16.17 | 0.71 | 0.006 |
| Systemic steroids | 28.31 | 30.29 | 30.79 | 32.28 | 31.91 | 33.01 | 33.47 | 35.37 | 36.32 | 0.89 | < 0.001 |
| **Other drugs** | | | | | | | | | | | |
| Leukotriene antagonists | 9.16 | 10.48 | 13.71 | 15.58 | 16.3 | 17.18 | 14.93 | 13.21 | 18.7 | 0.84 | 0.021 |
| Systemic decongestants | 51.66 | 53.24 | 52.53 | 52.53 | 54.79 | 55.38 | 56.17 | 56.91 | 55.79 | 0.63 | < 0.001 |
| Cough and cold preparations | 55.4 | 53.58 | 53.71 | 53.08 | 54.44 | 54.14 | 54.6 | 54.56 | 55.94 | 0.13 | 0.283 |
| **Prescription combination pattern** | | | | | | | | | | | |
| First-generation antihistamines only | 9.03 | 8.68 | 8.64 | 8.39 | 8.07 | 7.43 | 6.83 | 6.24 | 5.81 | -0.41 | < 0.001 |
| Second-generation antihistamines only | 51.6 | 51.38 | 51.81 | 51.23 | 50.51 | 50.15 | 49.11 | 47.58 | 48.55 | -0.5 | < 0.001 |
| Antihistamines and nasal steroids | 8.79 | 9.35 | 8.44 | 9.52 | 9.12 | 9.04 | 10.69 | 13.41 | 13.08 | 0.56 | 0.007 |
| Nasal steroids only | 1.99 | 2.03 | 1.88 | 2.07 | 2.13 | 2.11 | 2.56 | 3.17 | 3.09 | 0.15 | 0.003 |
| **First-choice prescription** | | | | | | | | | | | |
| Antihistamines only | 76.49 | 75.81 | 75.76 | 74.97 | 72.11 | 69.96 | 68.64 | 66.51 | 65.16 | -1.54 | < 0.001 |
| Nasal steroids only | 2.2 | 2.24 | 2.06 | 2.29 | 2.34 | 2.3 | 2.72 | 3.53 | 3.42 | 0.17 | 0.004 |
| Antihistamines and nasal steroids | 5.55 | 5.89 | 5.42 | 6.07 | 5.87 | 5.95 | 6.74 | 8.63 | 8.77 | 0.39 | 0.004 |
| Prescription and pattern rates per 100 patients are presented by year. Rates were sex- and age-standardized according to the population composition of 2010. | | | | | | | | | | | |

Supplemental Table 2e. Rates of prescription and patterns by year for age 40–64 years

| **Year** | **2010** | **2011** | **2012** | **2013** | **2014** | **2015** | **2016** | **2017** | **2018** | **Beta** | ***P*-value** |
| --- | --- | --- | --- | --- | --- | --- | --- | --- | --- | --- | --- |
| **Age 40-64 years** | | | | | | | | | | | |
| **Antihistamines** | | | | | | | | | | | |
| Total antihistamines | 85.16 | 85.5 | 84.97 | 85.01 | 82.16 | 81.18 | 80.68 | 80.57 | 79.91 | -0.8 | < 0.001 |
| First generation | 33 | 33.12 | 32.43 | 31.99 | 30.48 | 28.77 | 28.15 | 26.65 | 25.02 | -1.05 | < 0.001 |
| Second generation | 72.28 | 73.29 | 72.81 | 73.87 | 70.93 | 70.86 | 71.02 | 71.63 | 71.37 | -0.25 | 0.071 |
| **Steroids** | | | | | | | | | | | |
| Total steroids | 35.05 | 37.39 | 36.84 | 38.84 | 38.78 | 40.14 | 41.59 | 43.8 | 44.47 | 1.13 | < 0.001 |
| Nasal steroids | 9.86 | 10.72 | 10.27 | 10.83 | 10.51 | 10.72 | 12.33 | 15.37 | 14.96 | 0.64 | 0.004 |
| Systemic steroids | 28.37 | 30.55 | 30.21 | 31.9 | 32.25 | 33.39 | 33.82 | 34.21 | 34.85 | 0.76 | < 0.001 |
| **Other drugs** | | | | | | | | | | | |
| Leukotriene antagonists | 8.33 | 9.69 | 13.22 | 14.53 | 15.75 | 16.21 | 14.93 | 13.15 | 17.79 | 0.89 | 0.01 |
| Systemic decongestants | 43.69 | 45.04 | 43.58 | 43.55 | 47.27 | 47.12 | 48.13 | 48.34 | 47.11 | 0.6 | 0.006 |
| Cough and cold preparations | 56.02 | 53.24 | 53.67 | 52.8 | 53.5 | 52.29 | 52.74 | 52.6 | 53.61 | -0.23 | 0.104 |
| **Prescription combination pattern** | | | | | | | | | | | |
| First-generation antihistamines only | 12.49 | 11.7 | 11.66 | 10.61 | 10.79 | 9.81 | 9.23 | 8.43 | 8.04 | -0.55 | < 0.001 |
| Second-generation antihistamines only | 46.55 | 46.34 | 46.79 | 46.95 | 45.65 | 46.31 | 45.39 | 44.8 | 45.75 | -0.19 | 0.027 |
| Antihistamines and nasal steroids | 7.83 | 8.75 | 8.25 | 8.78 | 8.45 | 8.66 | 9.99 | 12.37 | 12.05 | 0.52 | 0.004 |
| Nasal steroids only | 2.03 | 1.97 | 2.02 | 2.06 | 2.06 | 2.07 | 2.34 | 3 | 2.92 | 0.12 | 0.006 |
| **First-choice prescription** | | | | | | | | | | | |
| Antihistamines only | 77.63 | 77.38 | 76.86 | 76.39 | 73.44 | 72.15 | 70.85 | 68.77 | 68.27 | -1.33 | < 0.001 |
| Nasal steroids only | 2.23 | 2.21 | 2.22 | 2.24 | 2.22 | 2.27 | 2.47 | 3.33 | 3.23 | 0.13 | 0.011 |
| Antihistamines and nasal steroids | 4.92 | 5.34 | 5.19 | 5.72 | 5.51 | 5.56 | 6.31 | 8.11 | 7.94 | 0.37 | 0.002 |
| Prescription and pattern rates per 100 patients are presented by year. Rates were sex- and age-standardized according to the population composition of 2010. | | | | | | | | | | | |

Supplemental Table 2f. Rates of prescription and patterns by year for age ≥65 years

| **Year** | **2010** | **2011** | **2012** | **2013** | **2014** | **2015** | **2016** | **2017** | **2018** | **Beta** | ***P*-value** |
| --- | --- | --- | --- | --- | --- | --- | --- | --- | --- | --- | --- |
| **Age ≥65 years** | | | | | | | | | | | |
| **Antihistamines** | | | | | | | | | | | |
| Total antihistamines | 85.26 | 85.43 | 85.06 | 84.19 | 81.55 | 80.15 | 81.37 | 80.74 | 80.93 | -0.71 | 0.001 |
| First generation | 41.15 | 41.37 | 41.92 | 40.94 | 36.84 | 35.03 | 35.17 | 33.56 | 33.39 | -1.23 | < 0.001 |
| Second generation | 67.12 | 67.17 | 67.53 | 66.99 | 66.01 | 65.7 | 67.28 | 67.49 | 68.15 | 0.06 | 0.607 |
| **Steroids** | | | | | | | | | | | |
| Total steroids | 26.76 | 28.57 | 28.65 | 30.27 | 30.39 | 31.31 | 33.05 | 33.93 | 34.64 | 0.96 | < 0.001 |
| Nasal steroids | 6.74 | 7.16 | 6.87 | 7.27 | 7.57 | 7.44 | 8.46 | 10.22 | 10.33 | 0.45 | 0.001 |
| Systemic steroids | 21.36 | 23.05 | 23.3 | 24.73 | 24.87 | 25.72 | 26.62 | 26.19 | 26.83 | 0.65 | < 0.001 |
| **Other drugs** | | | | | | | | | | | |
| Leukotriene antagonists | 6.94 | 8.06 | 11.17 | 12.48 | 13.03 | 13.47 | 12.56 | 10.84 | 13.96 | 0.67 | 0.019 |
| Systemic decongestants | 30.41 | 30.02 | 28.46 | 28.04 | 31.87 | 31.8 | 31.43 | 32 | 30.53 | 0.27 | 0.168 |
| Cough and cold preparations | 57.07 | 55.65 | 55.34 | 53.7 | 54.87 | 53.84 | 54.03 | 53.84 | 54.73 | -0.29 | 0.031 |
| **Prescription combination pattern** | | | | | | | | | | | |
| First-generation antihistamines only | 17.7 | 17.8 | 17.14 | 16.75 | 15.12 | 14.02 | 13.67 | 12.89 | 12.41 | -0.76 | < 0.001 |
| Second-generation antihistamines only | 40.71 | 40.64 | 40 | 39.9 | 41.06 | 41.59 | 42.07 | 41.61 | 42.17 | 0.24 | 0.01 |
| Antihistamines and nasal steroids | 4.95 | 5.25 | 5.05 | 5.25 | 5.52 | 5.46 | 6.16 | 7.76 | 7.64 | 0.35 | 0.002 |
| Nasal steroids only | 1.79 | 1.91 | 1.82 | 2.02 | 2.04 | 1.98 | 2.3 | 2.46 | 2.69 | 0.1 | < 0.001 |
| **First-choice prescription** | | | | | | | | | | | |
| Antihistamines only | 79.69 | 79.52 | 79.06 | 78.2 | 74.95 | 73.28 | 73.98 | 72.07 | 72.98 | -1.07 | < 0.001 |
| Nasal steroids only | 2.19 | 2.19 | 2.12 | 2.27 | 2.22 | 2.23 | 2.55 | 2.83 | 3.08 | 0.11 | 0.004 |
| Antihistamines and nasal steroids | 2.71 | 2.98 | 3.06 | 3.05 | 3.12 | 3.11 | 3.55 | 4.7 | 4.51 | 0.22 | 0.003 |
| Prescription and pattern rates per 100 patients are presented by year. Rates were sex- and age-standardized according to the population composition of 2010. | | | | | | | | | | | |

Supplemental Table 2g. Rates of prescription and patterns by year for allergic patients with atopic dermatitis

| **Year** | **2010** | **2011** | **2012** | **2013** | **2014** | **2015** | **2016** | **2017** | **2018** | **Beta** | ***P*-value** |
| --- | --- | --- | --- | --- | --- | --- | --- | --- | --- | --- | --- |
| **Atopic dermatitis** | | | | | | | | | | | |
| **Antihistamines** | | | | | | | | | | | |
| Total antihistamines | 84.46 | 85.03 | 84.5 | 84.29 | 83.55 | 82.68 | 82.45 | 82.46 | 81.68 | -0.41 | < 0.001 |
| First generation | 29.63 | 30.08 | 29.92 | 28.65 | 28.84 | 27.58 | 28.12 | 27.81 | 27.17 | -0.36 | < 0.001 |
| Second generation | 75.61 | 75.81 | 76.09 | 76.19 | 75.21 | 75.12 | 74.7 | 74.63 | 73.95 | -0.23 | 0.003 |
| **Steroids** | | | | | | | | | | | |
| Total steroids | 30.87 | 32.64 | 31.91 | 33.85 | 32.67 | 33.04 | 34.95 | 38.12 | 37.3 | 0.79 | 0.001 |
| Nasal steroids | 10.11 | 10.85 | 10.5 | 11 | 10.54 | 10.32 | 12.4 | 16.08 | 15.14 | 0.65 | 0.01 |
| Systemic steroids | 24.19 | 25.53 | 25.31 | 26.52 | 25.86 | 26.49 | 27.16 | 27.97 | 27.61 | 0.41 | < 0.001 |
| **Other drugs** | | | | | | | | | | | |
| Leukotriene antagonists | 26.25 | 28.97 | 34.83 | 39.34 | 41.49 | 43.2 | 40.82 | 39.11 | 45.91 | 2.08 | 0.002 |
| Systemic decongestants | 56.53 | 58.32 | 58.78 | 58.85 | 60.25 | 62.61 | 63.21 | 63.7 | 63.09 | 0.92 | < 0.001 |
| Cough and cold preparations | 66.58 | 64.34 | 64.15 | 64.37 | 65.64 | 65.55 | 66.15 | 66.46 | 67.5 | 0.25 | 0.089 |
| **Prescription combination pattern** | | | | | | | | | | | |
| First-generation antihistamines only | 8.51 | 8.81 | 8.07 | 7.79 | 8.04 | 7.25 | 7.5 | 7.35 | 7.23 | -0.19 | < 0.001 |
| Second-generation antihistamines only | 48.44 | 48.35 | 48.17 | 48.73 | 48.54 | 48.54 | 46.78 | 44.77 | 44.97 | -0.46 | 0.011 |
| Antihistamines and nasal steroids | 8.78 | 9.43 | 9.21 | 9.55 | 9.02 | 9.01 | 10.65 | 14.02 | 13.29 | 0.57 | 0.011 |
| Nasal steroids only | 1.34 | 1.43 | 1.29 | 1.44 | 1.52 | 1.32 | 1.75 | 2.06 | 1.86 | 0.08 | 0.009 |
| **First-choice prescription** | | | | | | | | | | | |
| Antihistamines only | 74.11 | 75.01 | 73.66 | 72.65 | 71.74 | 70.33 | 69.38 | 67.21 | 66.36 | -1.09 | < 0.001 |
| Nasal steroids only | 1.72 | 1.69 | 1.64 | 1.84 | 1.74 | 1.53 | 1.98 | 2.58 | 2.39 | 0.1 | 0.025 |
| Antihistamines and nasal steroids | 4.43 | 4.72 | 4.38 | 4.78 | 4.4 | 4.52 | 4.83 | 6.62 | 6.41 | 0.24 | 0.021 |
| Prescription and pattern rates per 100 patients are presented by year. Rates were sex- and age-standardized according to the population composition of 2010. | | | | | | | | | | | |

Supplemental Table 2h. Rates of prescription and patterns by year for allergic patients with asthma

| **Year** | **2010** | **2011** | **2012** | **2013** | **2014** | **2015** | **2016** | **2017** | **2018** | **Beta** | ***P*-value** |
| --- | --- | --- | --- | --- | --- | --- | --- | --- | --- | --- | --- |
| **Asthma** | | | | | | | | | | | |
| **Antihistamines** | | | | | | | | | | | |
| Total antihistamines | 84.48 | 84 | 83.69 | 83.52 | 82.71 | 82.76 | 83.32 | 82.65 | 81.62 | -0.28 | < 0.001 |
| First generation | 31.74 | 31.82 | 32.44 | 32.37 | 31.46 | 32.14 | 31.8 | 30.46 | 30.27 | -0.19 | 0.045 |
| Second generation | 73.83 | 74.3 | 74.41 | 74.06 | 73.58 | 73.56 | 74.92 | 73.99 | 73.05 | -0.06 | 0.44 |
| **Steroids** | | | | | | | | | | | |
| Total steroids | 26.7 | 27.48 | 27.85 | 28.27 | 27.56 | 27.9 | 30.05 | 32.21 | 32.96 | 0.72 | 0.002 |
| Nasal steroids | 8.72 | 9.55 | 8.75 | 9.19 | 8.61 | 8.29 | 10.29 | 13.34 | 13.04 | 0.51 | 0.024 |
| Systemic steroids | 20.42 | 20.71 | 21.71 | 21.82 | 21.54 | 22.44 | 23.17 | 23.13 | 24.06 | 0.42 | < 0.001 |
| **Other drugs** | | | | | | | | | | | |
| Leukotriene antagonists | 16.69 | 19.6 | 26.55 | 29.75 | 32.69 | 34.99 | 32.76 | 33.13 | 41.34 | 2.61 | < 0.001 |
| Systemic decongestants | 59.35 | 60.62 | 60.93 | 60.58 | 62.12 | 62.32 | 64.45 | 65.29 | 65.97 | 0.82 | < 0.001 |
| Cough and cold preparations | 62.49 | 61.44 | 59.64 | 59.92 | 61.37 | 60.73 | 61.8 | 62.22 | 62.89 | 0.15 | 0.328 |
| **Prescription combination pattern** | | | | | | | | | | | |
| First-generation antihistamines only | 10.19 | 9.22 | 8.92 | 9.05 | 8.79 | 8.81 | 8.1 | 8.19 | 8.1 | -0.22 | < 0.001 |
| Second-generation antihistamines only | 47.91 | 46.69 | 46.32 | 46 | 46.33 | 45.74 | 45.48 | 44.17 | 43.75 | -0.44 | < 0.001 |
| Antihistamines and nasal steroids | 7.39 | 8.14 | 7.46 | 7.84 | 7.43 | 7.34 | 8.84 | 11.67 | 11.19 | 0.47 | 0.018 |
| Nasal steroids only | 1.32 | 1.41 | 1.29 | 1.36 | 1.18 | 0.95 | 1.45 | 1.67 | 1.85 | 0.05 | 0.186 |
| **First-choice prescription** | | | | | | | | | | | |
| Antihistamines only | 75.68 | 74.38 | 73.52 | 73.05 | 71.39 | 71.16 | 70.88 | 68.26 | 67.48 | -0.97 | < 0.001 |
| Nasal steroids only | 1.62 | 1.73 | 1.54 | 1.72 | 1.45 | 1.12 | 1.63 | 2.07 | 2.28 | 0.05 | 0.237 |
| Antihistamines and nasal steroids | 3.63 | 3.75 | 3.54 | 3.63 | 3.52 | 3.58 | 3.82 | 5.4 | 4.78 | 0.17 | 0.039 |
| Prescription and pattern rates per 100 patients are presented by year. Rates were sex- and age-standardized according to the population composition of 2010. | | | | | | | | | | | |

Supplemental Table 3. General characteristics of the patients

| **Year**  **(n)** | **2010**  **(167,524)** | **2011**  **(172,961)** | **2012**  **(179,311)** | **2013**  **(182,395)** | **2014**  **(194,709)** | **2015**  **(192,414)** | **2016**  **(206,756)** | **2017**  **(209,704)** | **2018**  **(213,420)** |
| --- | --- | --- | --- | --- | --- | --- | --- | --- | --- |
| **Sex** |  |  |  |  |  |  |  |  |  |
| Female | 91,587 (54.7) | 93,707 (54.2) | 97,081 (54.1) | 98,087 (53.8) | 104,826 (53.8) | 103,069 (53.6) | 110,508 (53.4) | 112,040 (53.4) | 113,563 (53.2) |
| Male | 75,937 (45.3) | 79,254 (45.8) | 82,230 (45.9) | 84,308 (46.2) | 89,883 (46.2) | 89,345 (46.4) | 96,248 (46.6) | 97,664 (46.6) | 99,857 (46.8) |
| **Age groups** |  |  |  |  |  |  |  |  |  |
| 0–5 years | 23,435 (14.0) | 25,345 (14.7) | 28,751 (16.0) | 30,457 (16.7) | 31,966 (16.4) | 33,241 (17.3) | 35,865 (17.3) | 37,097 (17.7) | 37,331 (17.5) |
| 6–12 years | 24,434 (14.6) | 24,207 (14.0) | 24,108 (13.4) | 24,062 (13.2) | 25,514 (13.1) | 25,133 (13.1) | 27,293 (13.2) | 27,026 (12.9) | 27,805 (13.0) |
| 13–19 years | 17,617 (10.5) | 17,167 (9.9) | 16,863 (9.4) | 17,000 (9.3) | 17,902 (9.2) | 17,104 (8.9) | 17,651 (8.5) | 17,720 (8.5) | 17,275 (8.1) |
| 20–39 years | 44,496 (26.6) | 44,774 (25.9) | 45,221 (25.2) | 44,972 (24.7) | 47,359 (24.3) | 44,782 (23.3) | 47,936 (23.2) | 48,161 (23.0) | 48,656 (22.8) |
| 40–64 years | 44,379 (26.5) | 47,869 (27.7) | 49,274 (27.5) | 50,206 (27.5) | 54,519 (28.0) | 53,905 (28.0) | 58,019 (28.1) | 59,315 (28.3) | 60,467 (28.3) |
| ≥65 years | 13,163 (7.9) | 13,599 (7.9) | 15,094 (8.4) | 15,698 (8.6) | 17,449 (9.0) | 18,249 (9.5) | 19,992 (9.7) | 20,385 (9.7) | 21,886 (10.3) |
| **Allergic disease** | |  |  |  |  |  |  |  |  |
| Allergic asthma | 13,556 (8.1) | 15,395 (8.9) | 17,490 (9.8) | 18,001 (9.9) | 19,350 (9.9) | 18,903 (9.8) | 20,839 (10.1) | 19,396 (9.2) | 22,040 (10.3) |
| Allergic dermatitis | 15,635 (9.3) | 16,257 (9.4) | 17,020 (9.5) | 18,194 (10.0) | 18,987 (9.8) | 18,932 (9.8) | 20,749 (10.0) | 22,016 (10.5) | 22,171 (10.4) |
| The general characteristics of the patients are presented with numbers and percentages. The difference in distribution by year was significant for all basic characteristics (*P* < 0.001). | | | | | | | | | |
